# Responsive deep brain stimulation for the treatment of Tourette syndrome

**DOI:** 10.1038/s41598-024-57071-5

**Published:** 2024-03-18

**Authors:** Michael S. Okun, Jackson Cagle, Julieth Gomez, Dawn Bowers, Joshua Wong, Kelly D. Foote, Aysegul Gunduz

**Affiliations:** 1 Department of Neurology, Norman Fixel Institute for Neurological Diseases, University of Florida, 3011 SW Williston Rd, Gainesville, FL 32608 USA; 2 Department of Neurosurgery, Norman Fixel Institute for Neurological Diseases, University of Florida, Gainesville, FL USA; 3 Department of Biomedical Engineering, Norman Fixel Institute for Neurological Diseases, University of Florida, Gainesville, FL USA; 4 Department of Clinical and Health Psychology, Norman Fixel Institute for Neurological Diseases, University of Florida, Gainesville, FL USA

**Keywords:** Neuromodulation, Tic, DBS, Closed loop, Programming, Selection, Neural circuits, Neuroscience, Neurology, Neurological disorders

## Abstract

To report the results of ‘responsive’ deep brain stimulation (DBS) for Tourette syndrome (TS) in a National Institutes of Health funded experimental cohort. The use of ‘brain derived physiology’ as a method to trigger DBS devices to deliver trains of electrical stimulation is a proposed approach to address the paroxysmal motor and vocal tic symptoms which appear as part of TS. Ten subjects underwent bilateral staged DBS surgery and each was implanted with bilateral centromedian thalamic (CM) region DBS leads and bilateral M1 region cortical strips. A series of identical experiments and data collections were conducted on three groups of consecutively recruited subjects. Group 1 (n = 2) underwent acute responsive DBS using deep and superficial leads. Group 2 (n = 4) underwent chronic responsive DBS using deep and superficial leads. Group 3 (n = 4) underwent responsive DBS using only the deep leads. The primary outcome measure for each of the 8 subjects with chronic responsive DBS was calculated as the pre-operative baseline Yale Global Tic Severity Scale (YGTSS) motor subscore compared to the 6 month embedded responsive DBS setting. A responder for the study was defined as any subject manifesting a ≥ 30 points improvement on the YGTSS motor subscale. The videotaped Modified Rush Tic Rating Scale (MRVTRS) was a secondary outcome. Outcomes were collected at 6 months across three different device states: no stimulation, conventional open-loop stimulation, and embedded responsive stimulation. The experience programming each of the groups and the methods applied for programming were captured. There were 10 medication refractory TS subjects enrolled in the study (5 male and 5 female) and 4/8 (50%) in the chronic responsive eligible cohort met the primary outcome manifesting a reduction of the YGTSS motor scale of ≥ 30% when on responsive DBS settings. Proof of concept for the use of responsive stimulation was observed in all three groups (acute responsive, cortically triggered and deep DBS leads only). The responsive approach was safe and well tolerated. TS power spectral changes associated with tics occurred consistently in the low frequency 2–10 Hz delta-theta-low alpha oscillation range. The study highlighted the variety of programming strategies which were employed to achieve responsive DBS and those used to overcome stimulation induced artifacts. Proof of concept was also established for a single DBS lead triggering bi-hemispheric delivery of therapeutic stimulation. Responsive DBS was applied to treat TS related motor and vocal tics through the application of three different experimental paradigms. The approach was safe and effective in a subset of individuals. The use of different devices in this study was not aimed at making between device comparisons, but rather, the study was adapted to the current state of the art in technology. Overall, four of the chronic responsive eligible subjects met the primary outcome variable for clinical effectiveness. Cortical physiology was used to trigger responsive DBS when therapy was limited by stimulation induced artifacts.

## Introduction

Deep brain stimulation (DBS) has received full Food and Drug Administration approval for essential tremor and Parkinson’s disease and an Humanitarian Device Exemption approval for dystonia and obsessive compulsive disorder. DBS research has recently been expanding to include other neuropsychiatric indications^1–5^. Approved indications, with the exception of epilepsy, have focused on the continuous delivery of electrical stimulation. Feedback-loop based systems capable of detecting ‘pathological brain physiology’ have been proposed as attractive alternatives^6–8^.

Our laboratory initiated a series of National Institutes of Health (NIH) funded experiments in 2006 to develop an early ‘responsive’ DBS system capable of suppressing Tourette syndrome (TS) related motor and vocal tics^9–14^. The system was designed to be triggered by tic-related brain physiology to address the hallmark paroxysmal tics of TS^14^. Further, we opined that if physiological changes could be associated with the occurrence of tics, we could feed this information into the design of a responsive DBS paradigm.

Our group has previously demonstrated that a marker for tics was present in human basal ganglia electrophysiological signals which were collected in the operating room and in the clinic settings^9–11,14^. In the current study, we provide data which was derived from a prospective consecutive experimental series including ten medication refractory TS subjects. This single cohort underwent the implantation of bi-directional DBS devices which were capable of both sensing physiology and stimulating the target through the same DBS lead. The aim of the study was to demonstrate the safety and proof of concept for effectiveness of a responsive DBS approach to reduce motor and vocal tics in the setting of TS. Each subject underwent implantation of bilateral centromedian nucleus (CM) region DBS and bilateral primary motor cortex (M1) motor strip implantations. We tested acute responsive DBS using both deep and superficial leads (n = 2), chronic responsive DBS using deep and superficial leads (n = 4) and responsive DBS using only the deep leads (n = 4). We collected the primary effectiveness outcome for individual subjects and we also calculated a combined responder rate for the responsive cohort. We collected and summarized the individualized programming methodologies applied for each participant to enable a TS responsive DBS approach. We compiled a summary of subject specific physiology enabling a responsive TS DBS approach.

## Methods

### Participants

TS subjects were recruited from the Fixel Institute for Neurological Diseases Campus at the University of Florida. The inclusion criteria were previously published and can be summarized by^11^ (1) a minimum of 21 years old with a diagnosis of TS based on DSM-V criteria, (2) a total tic score based on the YGTSS greater than 35 points of a possible 50, (3) a history of trials of multiple classes of tic medications with evidence of lack of effectiveness in managing tic symptoms, (4) offered botulinum toxin injections if focal tics were present, and (5) offered cognitive behavioral interventional therapy.

Ten subjects signed informed consent (IRB 201300850) and met FDA and Investigational Device Exemption (IDE) criteria and were recruited for the study (G130253). (Clinicaltrials.gov registration NCT02056873, registered 06/02/2014; first subject enrolled 20/03/14, trial completed 30/06/2023). Each subject underwent multidisciplinary review prior to consideration of an implant (neurology, psychiatry, neurosurgery). There was also an independent ethics review conducted by Dr. William Allen an ethicist at the University of Florida.

**Figure 1:**
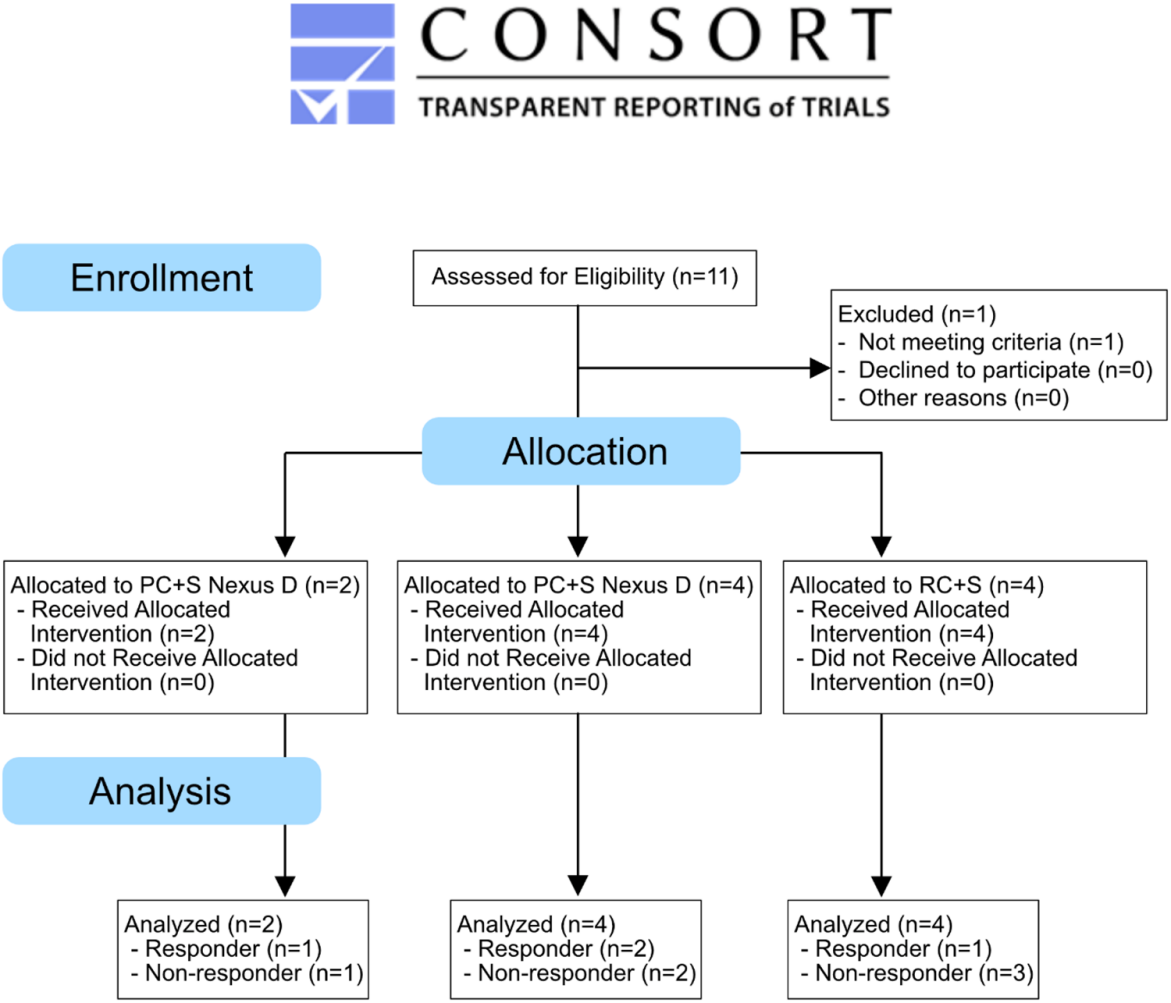
CONSORT Diagram summarizing the study design.

The CONSORT diagram for the study design is provided in Fig. 1. All the methods were carried out in accordance with institutional guidelines and regulations. All experimental protocols were approved by the IRB and FDA.

All subjects underwent bilateral staged DBS surgery. Each subject was implanted with bilateral CM region DBS leads and bilateral M1 cortical strip leads. The cortical strips were placed over the hand motor cortices or alternatively could be adjusted by the neurosurgeon to better align with body parts most affected by tics. Strip placement was limited to the size of the electrodes and the size of what could be positioned through a burr hole. The cortical leads were used for sensing but not used for stimulation. Thirty days following lead implantations, the neurostimulator(s) were subclavicularly implanted in a separate operation.

### Devices

The study used first generation bidirectional DBS devices, which included a sensing engine to access neural signals, a classifier to detect pathological signals, and finally a stimulation engine. The first two subjects received the Activa PC + S (Medtronic, Minneapolis) neurostimulators without chronic embedded responsive DBS capabilities. Responsive DBS for these two subjects was tested using the Nexus-D system (Medtronic, Minneapolis). Nexus-D is a telemetry interface that facilitates communication between the neurostimulator and a computer. Nexus-D enables streaming of neural data onto a computer, and the computer transfers commands to the stimulation engine^15^. The next four subjects enrolled in the study had Activa PC + S DBS systems with a firmware upgrade, known as Nexus-E, which facilitated the sensing engine’s classifier output and was capable of directly communicating with the stimulation engine. This upgrade enabled chronic embedded responsive DBS therapy^16^. The final four subjects in the study received a new second generation bidirectional neurostimulator, the Summit RC + S system (Medtronic, Minneapolis), which supported embedded responsive DBS. The Summit RC + S was rechargeable and the system was capable of delivering active biphasic stimulation pulses which were employed as a method to reduce stimulation artifacts.

### Application of responsive stimulation

**Figure 2:**
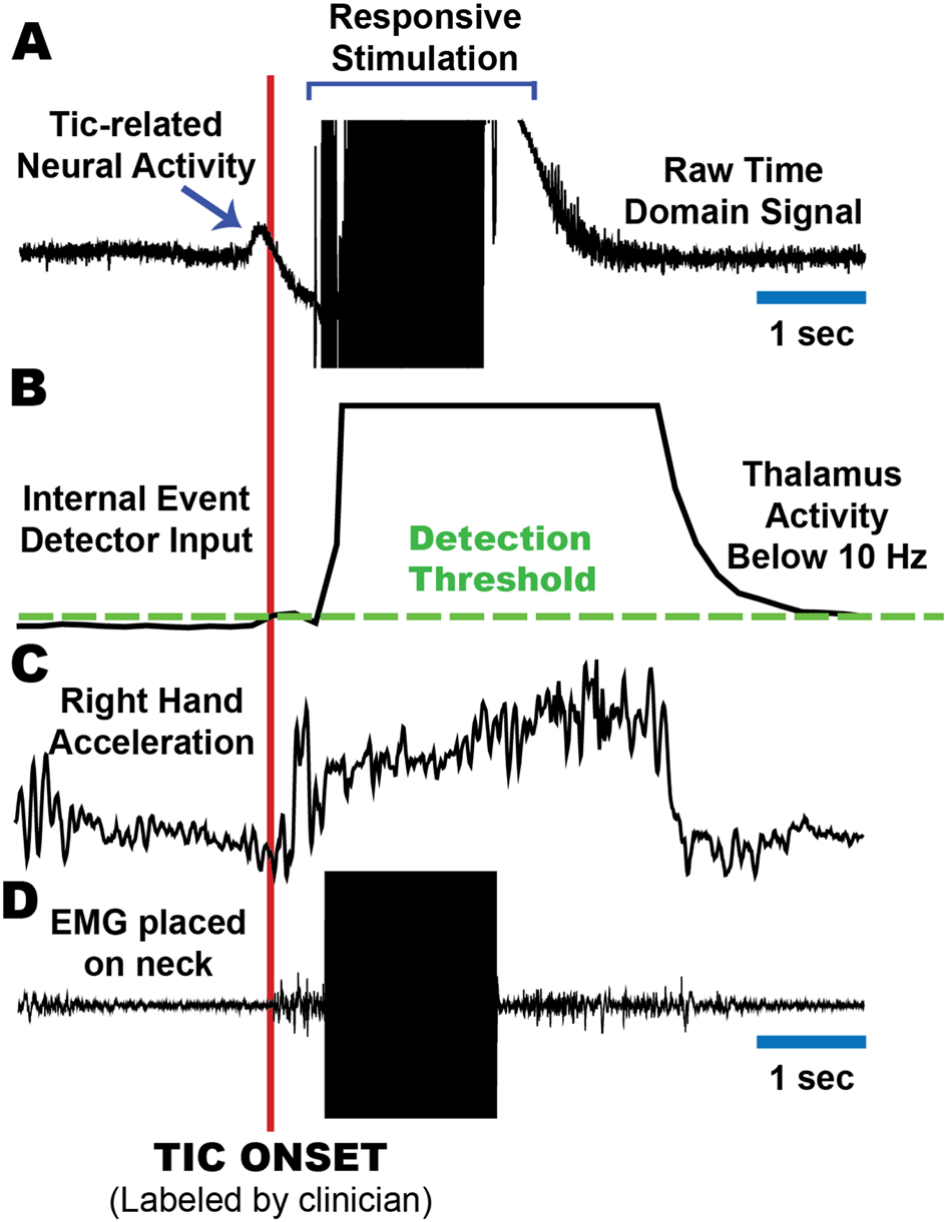
The use of the neural marker to trigger responsive deep brain stimulation. Time domains signals from multiple modalities aligned to the onset of tic (red line). Brain signals are streamed to a computer via Nexus-D. (**A**) Time domain signal from the CM thalamus. (**B**) The low frequency power in the signal that is inputted to the tic detector (threshold in green). (**C**) Acceleration of hand affected by tic. We can observe that the neuromarker appears prior to the motor tic execution. (**D**) The EMG/inertia system used to align all signals. Stimulation artifact observed in the brain signal can also be captured by an EMG signal placed on the neck, where the connector cables are tunneled.

PC + S devices have an onboard linear discriminant analysis (LDA) classifier that initiates or terminates stimulation based on the spectral power of neural signals. This classifier requires programming of symptom neuromarkers. Both thalamic and motor cortex signals were analyzed to identify the spectral features of tics. When using Nexus-D, the classifier output was sent to a computer which initiated CM stimulation when tic physiology was detected. The computer closed the loop in order to create responsive neurostimulation paradigm. This type of system was useful for in clinic prototyping and for development of a fully embedded closed-loop system. (Fig. 2). Only subjects 1–2 used the Nexus-D system.

The Nexus-E upgrade (subjects 3–6) facilitated the use of a classifier output which could communicate directly with the stimulation engine and could enable embedded chronic responsive therapy. The Nexus-E firmware’s spectral power computation was low resolution (10-bit) and was prone to be influenced by stimulation artifacts.

If the physiology and clinical tic features in an individual subject manifested unilaterally, a different detection and stimulation paradigm was employed. One neurostimulator was used to identify tic related signals, while the other neurostimulator which was programmed at an offset stimulation frequency, detected the changes in stimulation state of the first neurostimulator and automatically triggered the DBS therapy to be deployed to the contralateral hemisphere.

The final hardware used in the study was the Summit RC + S (Medtronic, Minneapolis) and the technology was based on a similar architecture of the PC + S. The upgraded design was able to more efficiently suppress stimulation artifacts when using the same DBS lead which facilitated the device to be used for both feature detection and for stimulation. Subjects 7–10 received both acute in clinic and chronic responsive DBS through the RC + S device. Subject 7 received responsive DBS at 6 months, Subjects 8 and 9 did not receive responsive DBS, and Subject 10 had a trial of responsive DBS at 24 months.

In both hemispheric devices, during quiescent periods of the embedded responsive therapy, the device could be set to be deactivated or alternatively could be set to a lower stimulation level. It was never set to 0 mA. A chronic low stimulation level setting was utilized in all subjects to reduce ramping time and to improve tolerability. When the classifier detected neural signatures of tic, stimulation was incremented at a clinician-defined rate until reaching a therapeutic stimulation level. Stimulation could be delivered with a fixed duration (subjects 3–6), or could be guided by the pathological tic signal if it was not obscured by artifacts (subjects 7–10).

### Clinical programming

Each clinician who programmed the DBS devices documented improvements or worsening in tics, decreases or increases in anxiety and each clinician used the presence and absence of stimulation induced adverse effects as a guide for device optimization. When tics were reported to improve, the settings were empirically adjusted using longer pulse widths, increased frequencies and/or higher milliamps to optimize the benefit without adjusting the location of the active stimulation contacts. If no improvement in tics was reported, the four possible stimulation contacts on the DBS lead were empirically changed by the clinician. If no, or mild benefit was reported, the stimulation mode was trialed in a monopolar and in a bipolar configuration. The responsive DBS setting was selected based on the optimal “open-loop” continuous DBS setting. The initial programming settings were provided by the clinical programmer following the first month visit, where a stimulation sweep was performed across all contacts. The contact with the most immediate response and largest therapeutic window (high tolerability of stimulation) was chosen as the initial contact. A secondary contact, or bipolar stimulation, was only be chosen if no clinical responses were observed after 1–2 months of continuous therapy. The contact selection was purely clinically driven without input from the neurophysiology team.

Subjects were only switched to responsive stimulation chronically, if open-loop settings were considered optimized. During each programming visit, subjects were examined by a psychiatrist using the Yale Global Tic Severity Scale (YGTSS) and the Modified Rush Videotape Rating Scale for Tic (MRVRST). These visits included collection of a validated score of the clinical effects which was drawn from the prior months DBS settings. The primary clinical outcome measure for this pilot study was the comparison of pre-operative baseline YGTSS to continuous DBS and embedded responsive DBS settings for each participant (e.g. to be deemed a responder each subject had to meet the criteria). The videotaped MRVTRS was recorded as a secondary outcome. The outcomes were collected 6 months after the initial implants. For the MRVRST, during the primary outcome visit, the psychiatrist recorded three sets of video recordings with three different stimulation paradigms: no stimulation, conventional open-loop stimulation, and embedded responsive stimulation. The order of the stimulation mode was randomized for each subject. Neither the psychiatrist nor the subjects were aware of the type of stimulation which was administered when scoring. The scores were cross-compared using one-way analysis of variance (ANOVA), and then by post-hoc comparison using a Wilcoxon signed-rank test between baseline, open-loop, and embedded responsive conditions. Secondary outcomes included the Hamilton Depression Scale, the Young Mania Rating Scale, the Conners’ Adult ADHD Rating Scale, the Yale Brown Obsessive Compulsive Scale and the MOS-36 Quality of life.

### Approval statement

The University of Florida Institutional Review board reviewed and approved IRB 201300850. The FDA also reviewed the proposal and provided an Investigational Device Exemption (IDE) (G130253). The ethics for the study were overseen by Dr. William Allen an ethicist on faculty at the University of Florida.

## Results

### Cohort description

The mean age of the 10 subjects in the TS cohort at the time of surgery was 30.1 ± 8.2 and the mean disease duration was 22.6 ± 6.9 years (Table 1). There were 5 males and 5 females in the cohort.

**Table 1:** Tourette DBS cohort characteristics.

Subject	Age of tic onset	Age at surgery	Gender	Responsive DBS implementation
1	8	23	F	Y (Acute)
2	8	25	F	Y (Acute)
3	8	33	F	Y
4	12	39	M	Y
5	7	26	F	Y
6	11	45	M	Y^a^
7	6	24	M	Y
8	5	24	M	Y
9	4	21	M	N^b^
10	6	41	F	Y

^a^Responsive DBS tested at the 6 month outcome, ^b^double negative bipolar was an open loop setting which could not be used for closed loop testing.

**Figure 3:**
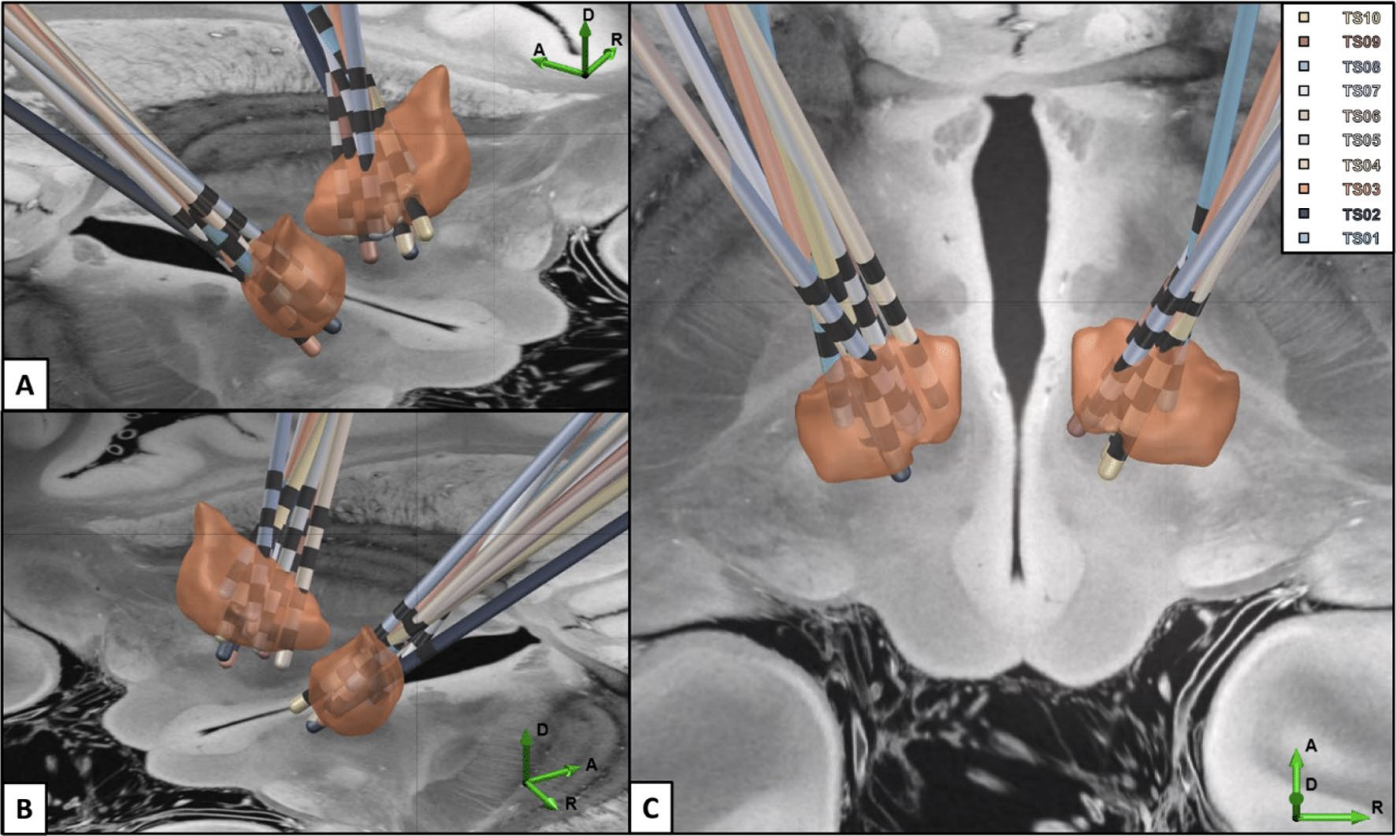
Locations of the Tourette deep brain stimulation leads. Lead DBS software was used to reconstruct the lead locations for the each of the subjects in this study and they are displayed in several axial representations (**A**–**C**).

A series of identical experiments and data collections were conducted in all three groups of consecutively recruited subjects. Group 1 (n = 2) used acute responsive DBS using deep and superficial leads. Group 2 (n = 4) used chronic responsive DBS using deep and superficial leads. Group 3 (n = 4) used responsive DBS with only deep leads. Figure 3 summarizes the lead locations reconstructed using LEAD DBS for the ten subjects in the study.

### Results of chronic responsive deep brain stimulation

The outcomes data for all 10 subjects in the study is provided in Table 2. Eight subjects in the study had devices capable of chronic responsive DBS (subjects 2–10). Four of the 8 subjects in the cohort (50%) met the pre-defined primary outcome change of > 30 point change on the YGTSS motor scale (Table 3). There was a 43.5% improvement in motor tics and a 62.2% improvement in vocal tics. There was also a YGTSS global impression improvement of 62.5%. The secondary outcome of the MRVRST improved 63.8% (Table 2). Since the YGTSS is measured over the prior weeks of therapy it was impossible to compare continuous DBS to responsive DBS at the 6 month primary outcome variable.

**Table 2:** Outcomes for all TS DBS subjects.

Subject	YGTSS—motor score		YGTSS—vocal score		YGTSS global impression		MRVRST—open		MRVRST—closed	
	Baseline	6 Month	Baseline	6 Month	Baseline	6 Month	Baseline	6 Month	Baseline	6 Month
TS01	25	18	20	16	50	30	20	13	20	N/A
TS02	21	14	23	13	40	30	17	8	17	N/A
TS03	20	11	22	11	40	20	17	8	17	5
TS04	21	17	20	10	40	30	11	8	11	6
TS05	20	6	21	8	40	0	10	3	10	3
TS06	18	24	17	23	30	40	12	9	12	7
TS07	25	15	23	3	40	10	13	N/A	13	4
TS08	23	25	17	12	40	40	N/A	15	N/A	N/A
TS09	23	21	22	16	40	40	16	14	16	N/A
TS10	19	16	19	17	30	30	11	10	11	N/A
All subjects		− 21.78%		− 34.43%		− 28.17%		− 35.59%		− 59.39%

**Table 3:** Responder outcomes for TS responsive deep brain stimulation.

Patient ID	YGTSS—motor score		YGTSS–vocal score		YGTSS global impression		MRVRST—closed loop	
	Baseline	6 Month	Baseline	6 Month	Baseline	6 Month	Baseline	6 Month
TS03	20	11	22	11	40	20	17	5
TS04	21	17	20	10	40	30	11	6
TS05	20	6	21	8	40	0	10	3
TS07	25	15	23	3	40	10	13	4
Percent improvement		43.51%		62.22%		62.50%		63.82%

The abbreviations in the table: Yale Global Tic Severity Scale (YGTSS) and the Modified Rush Videotape Rating Scale for Tic (MRVRST). Closed loop refers to the responsive DBS condition.

### Results for the cohort receiving chronic continuous deep brain stimulation

There were 2 subjects with devices which were not enabled for responsive DBS and 4 subjects who had enabled devices however were stimulated in open loop chronic continuous DBS settings. Tourette subjects 6, 8, 9 and 10 were considered non-responders to responsive DBS. Across this cohort the YGTSS motor scores improved by 7.3% the vocal scores by 15.9% and the global impression score by 5.3%. There was a 15.5% improvement in the videotaped MRVRST however the two acute subjects (#1 and 2) did not have MRVRST scales (Table 4).

**Table 4:** Outcomes for cohort on chronic continuous DBS.

Patient ID	YGTSS—motor score		YGTSS—vocal score		YGTSS global impression		MRVRST—open	
	Baseline	6 Month	Baseline	6 Month	Baseline	6 Month	Baseline	6 Month
TS01	25	18	20	16	50	30	20	13
TS02	21	14	23	13	40	30	17	8
TS06	18	24	17	23	30	40	12	9
TS08	23	25	17	12	40	40	N/A	15
TS09	23	21	22	16	40	40	16	14
TS10	19	16	19	17	30	30	11	10
All		7.30%		15.90%		5.28%		15.53%

YGTSS, Yale Global Tic Severity Score; MRTRS, Modified Rush Tic Rating Scale Score.

### Secondary behavioral and mood outcomes

There were no significant changes in the Hamilton Depression Scale, the Young Mania Rating Scale, the Conners’ Adult ADHD Rating Scale, and the Yale Brown Obsessive Compulsive Scale when comparing baseline to 6 month outcomes (Table 5). Notably, on the CAARS scale only 5 subjects had a score of > 60 indicating clinically relevant ADHD at baseline. Two of those subjects worsened and 3 improved.

**Table 5:** Depression, Mania, attention deficit and hyperactivity and obsessive compulsive outcomes.

Subjects	HAM-D		YMRS		CAARS		Y-BOCS	
	Baseline	6 Month	Baseline	6 Month	Baseline	6 Month	Baseline	6 Month
TS01	2	12	0	7	N/A	66	7	8
TS02	3	6	0	6	74	97	0	5
TS03	4	9	2	0	19	69	0	0
TS04	4	N/A	3	0	89	59	28	22
TS05	7	1	1	0	56	40	0	0
TS06	0	1	0	4	62	59	0	0
TS07	8	0	0	0	71	54	0	0
TS08	8	11	0	0	140	126	32	9
TS09	9	0	0	0	15	31	0	18
TS10	N/A	N/A	N/A	N/A	N/A	N/A	N/A	N/A
Percent improvement		0.13		1.22		1.13		0.56

HAM-D, Hamilton Depression Scale; YMRS, Young Mania Rating Scale; CAARS, Conners’ Adult ADHD Rating Scale; Y-BOCS, Yale Brown Obsessive Compulsive Scale (Y-BOCS).

### Quality of life outcomes

The MOS Short Form Survey (MOS-36) outcomes comparing baseline to 6 months revealed > 10 point improvements in physical functioning, role limitation due to physical health, role limitation due to emotional problems, social functioning, pain and health change. However when controlling for multiple comparisons, the group changes were not significant (Table 6).

**Table 6:** Quality of life outcomes.

Subjects	Physical function		Role limitation due to physical health		Role limitation due to emotional problem		Energy/Fatigue		Emotional wellbeing		Social functioning		Pain		General health		Health change	
	Baseline	6 Month	Baseline	6 Month	Baseline	6 Month	Baseline	6 Month	Baseline	6 Month	Baseline	6 Month	Baseline	6 Month	Baseline	6 Month	Baseline	6 Month
TS01	35	20	0	0	100	100	0	0	76	52	0	12.5	0	10	25	10	0	50
TS02	95	80	100	100	100	66.7	55	40	80	64	100	75	57.5	100	85	75	100	75
TS03	100	100	50	100	100	100	55	45	88	64	50	75	35	67.5	75	50	50	75
TS04	75	N/A	75	N/A	100	N/A	40	N/A	96	N/A	87.5	N/A	100	N/A	65	N/A	50	N/A
TS05	85	100	100	100	33.3	100	20	55	52	76	37.5	100	45	100	80	80	50	75
TS06	100	100	100	100	33.3	100	95	50	76	72	87.5	100	67.5	100	90	70	50	50
TS07	100	100	100	100	33.3	100	35	20	80	76	87.5	100	100	90	75	50	25	50
TS08	30	100	0	100	0	66.7	15	60	40	44	25	37.5	10	80	70	45	50	50
TS09	55	100	25	100	100	100	10	20	72	88	12.5	100	80	90	95	100	50	50
TS10	N/A	N/A	N/A	N/A	N/A	N/A	N/A	N/A	N/A	N/A	N/A	N/A	N/A	N/A	N/A	N/A	N/A	N/A

The MOS Short Form Survey (MOS-36) was used to assess quality of life in each subject enrolled in the study and t-tests correcting for multiple comparisons revealed no significant changes.

### Physiology features for responsive DBS and ramp dynamics

**Figure 4:**
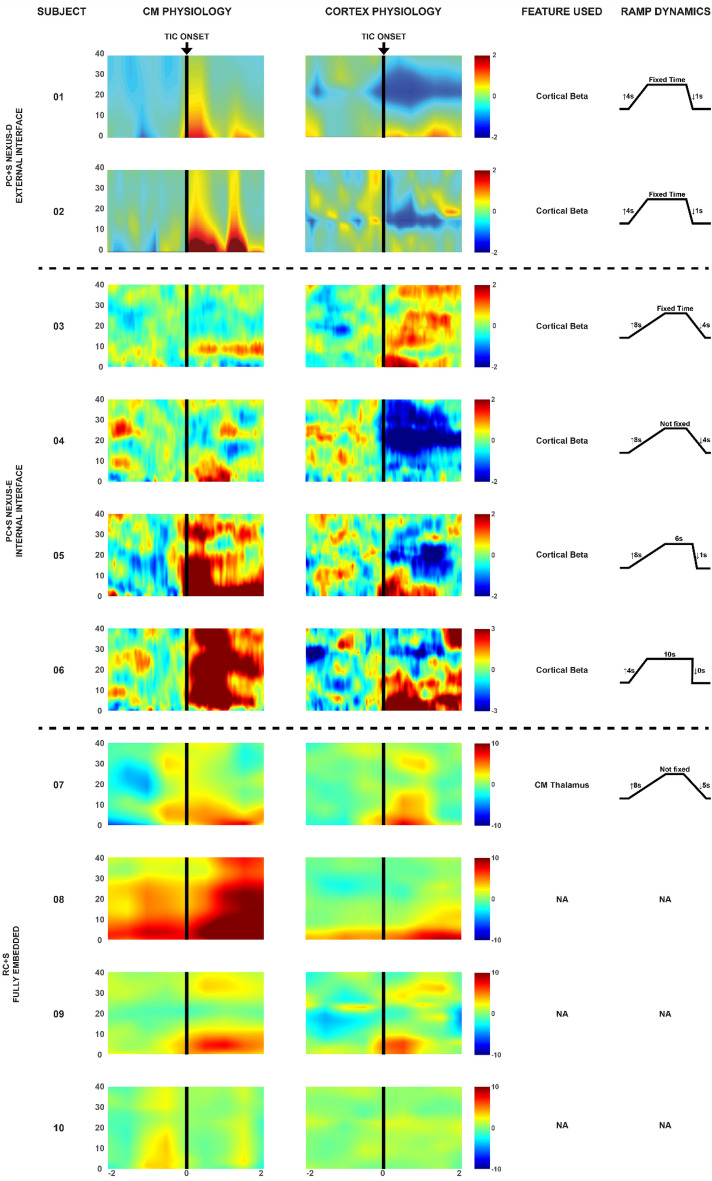
Physiology features for responsive deep brain stimulation and programming ramp dynamics. The electrophysiology represent the average spectrograms of the individual signals from each subject. X axis is time (seconds) and y axis is frequency (Hz). Tic onset is denoted by the arrow and black vertical line The feature used was the feature selected for responsive stimulation trials and the ramp on and off time were the values used to program the DBS device in responsive mode.

A summary of the CM physiology, the cortical physiology, the tic onset, the features selected for trials of responsive DBS and the ramp dynamics of the settings selected have been summarized in Fig. 4.

### Safety

One subject was explanted and reimplanted secondary to infection (Subject #8). One cortical lead was not replaced in this subject due to cortical scarring from the first implantation (Subject #8). A second subject had the deep DBS leads repositioned due to suboptimal lead locations (Subject #4). See supplementary Table 1 and 2 for a summary of all adverse events.

## Discussion

The results of this trial revealed that 4/8 (50%) of the refractory TS subjects met the primary outcome of a motor tic score reduction of ≥ 30% during chronic responsive DBS. The data suggested that for these 8 subjects, the approach employed was feasible. Additionally, when stimulation induced artifacts were encountered cortical beta power was used to trigger the responsive paradigm. Two of the subjects in the study used an externally triggered responsive DBS strategy and four used the chronic embedded DBS system. Across subjects TS power spectrum changes associated with tics occurred in the low frequency 2–10 Hz delta-theta-low alpha oscillation range. Subject #4 data revealed a correlation of premonitory urges to cortical beta power decreases and there was no associated thalamic beta change. The beta power decrease was used as a control signal for the application of chronic embedded responsive therapy. In subject #5 who had predominantly vocal tics, we observed a cortical beta power decrease, and this feature was used to implement chronic responsive stimulation. Another innovation introduced in this study was the use of one CM region DBS device to sense a tic and then serve as a trigger for bi-hemispheric delivery of therapeutic stimulation^17^. Finally, and most promising, improvements in both hardware and technology in the final 4 subjects led to the use of the CM DBS leads for both sensing and stimulation without the previous electrical artifact induced limitations. This study thus revealed the possibility that a single lead responsive DBS approach may be possible in TS.

Our TS DBS cohort will likely be useful for guiding a translatable approach for the implementation of responsive TS DBS technology. The first step we employed in programming was to identify tic features, especially those associated with a dominant or disabling tic. Next, we employed a process for aligning and labeling tics that were associated with brain physiology, using both video and motion sensors. Once labeled, tics of interest were analyzed by computing changes in spectral power and these were compared to baseline tic-free epochs. The DBS hardware employed in the study limited the choice of stimulation contact to the ‘middle’ contacts on each DBS lead, with the implementation of bipolar sensing electrode arrays set up on either side of the stimulation contact; also referred to as a ‘sandwich configuration’. Alternatively, monopolar DBS configurations could be employed but only if recording contacts were not used for stimulation. The choice of configuration was important for the limitation of side effects. The combination of avoiding stimulation induced side effects, and the use of a sandwich configuration can in some cases result in the use of a suboptimal contact for delivery of the electrical stimulation. The next step in programming was to determine the ramp tolerability. A manual closed-loop trigger test was used to determine whether an individual stimulation setting was associated with a side effect. The clinician used this information to choose and to test a ramp time. Once the ramp parameters were optimized, the clinician deployed the closed loop algorithm, using one or more features and by setting thresholds for detection of the tic feature. Once setup, DBS programming was an iterative process, taking weeks to months to empirically adjust thresholds, to monitor for side effects, to optimize models for tic detection and to suppress vocal and motor tics.

The identification of the physiology underpinning tics will remain a rate limiting step for the successful implementation of a responsive DBS TS therapy. Past work from our laboratory, and from other expert groups, has revealed that duty cycle DBS and continuous DBS are options when open or responsive loop paradigms may not be practically implemented^18^. Our previous experience using duty cycle DBS revealed that tics could be suppressed despite a device being inactivated for the majority of a 24-h period^13,18^. In the current study, though the device was programmed to be triggered by tic related activity, we hypothesized that the longer lasting tic suppression would be more likely to be related to effects on the neural network, however more data will be required to clarify this point.

This cohort was important for defining the underlying physiology of human tic. We aimed to use electrophysiology to differentiate tic from voluntary movement. Our first proof of concept for a responsive DBS device in TS was deployed in a single subject (2017) with a different device than used the current study (Neuropace, Mountainview, CA)^13^. In that subject, we used bilateral CM region DBS and we found that the device could sense and deliver DBS without artifact generation. Unfortunately, we could not replicate this capability with the newer hardware until the final four subjects in our current study, who had more advanced technology implanted. One critical observation, previously published from this cohort, was the more formal characterization of human tic physiology derived from the CM region^19^. Collectively, we learned that the CM region proved the preferable target for differentiating tic from voluntary movement, when we compared the physiology to that derived from the motor cortex. We were consistently able to identify tic as associated with low frequency (1–10 Hz) power increases in the CM region. We further observed that the occurrence of a tic was not associated with a typical oscillation, but rather was more commonly associated with a small evoked potential that occurred within 100–200 ms of a motor or vocal tic^11,14,19^. Finally, we were able to sample the tic physiology and to align the physiology with electromyogram recordings and the videotaped analyses to confirm baseline human tic physiology.

Within the cohort, only the final 4 subjects could be successfully programmed to both record and to stimulate from the same DBS lead without the induction a limiting stimulation induced artifact. Adverse events and surgical morbidity from this procedure will likely be decreased if we can develop a consistent single sense and stimulation paradigm which could avoid unnecessary ‘sense only’ hardware.

The results from this study demonstrated the potential to safely apply chronically embedded DBS hardware into multiple brain targets, beyond a standard approach, which is commonly limited to two DBS leads. Our approach was to implant deep leads bilaterally in the thalamus along with superficial leads placed over the M1 motor cortex. Unfortunately, due to technology limitations, our first 2 subjects had only acute closed loop DBS. These 2 subjects did however, provide the information necessary for the refinement of a ‘sense from one brain target and stimulate from another brain target’ paradigm. This paradigm, though requiring two extra cortical DBS leads, overcame the serious issue of stimulation related artifacts. We utilized data from the next four subjects from this cohort (subjects 3–6) to show that when recording from leads located in CM versus the motor cortex, tic was more precisely differentiated from voluntary movement^19^. The cortical DBS leads were thus critically important for these 4 TS DBS subjects. The final 4 subjects in the cohort were implanted with technology which facilitated recording and stimulation from the same CM region DBS lead, thus eliminating the need for the cortical strip.

There were important technical and patient selection issues which emerged in the final 4 subjects. The rechargeable battery technology proved troublesome for two subjects, and this resulted in the unintended automatic reset of the adaptive stimulation settings to the device factory default settings. Additionally, the data from this study will be most informative to the CM DBS target and not the aGPi or other emerging TS DBS applications. One major limitation was that ramp time was purposely slow in order to avoid adverse effects. This slow ramp time prevented continuous responsive stimulation, especially during flurries of tics. One important observation from many years of work in TS DBS is that responsive stimulation may be effective as a result of ‘resetting of the neural network.’ It may not be necessary for the device to detect every tic event in this paradigm, and this may explain why outcomes tend to be better over time. Though not tracked in this cohort it is possible that we will learn that responsive paradigms in Tourette have obvious advantages in battery consumption and possible in reduction of side effects. A final limitation for the group who used cortical sensing was that although they had similar effectiveness there may have been slightly more overstimulation due to feature overlap between tic and movement.

In summary, this study revealed that tic related physiology enabled the potential in some participants for responsive TS DBS. Overall, four of the subjects enrolled in the study met the primary outcome. TS power spectrum changes associated with tics occurred consistently in the low frequency 2–10 Hz delta-theta-low alpha oscillation range. The use of different devices in this study was not aimed at making between device comparisons, but rather, the study was adapted to the current state of the art in technology. Cortical physiology proved a useful marker to trigger responsive DBS especially when therapy was limited by stimulation induced artifacts. The novel use of one hemispheric device sensing a tic and then triggering both bilateral devices to deliver a train of stimulation could be important for use in future studies. Our data revealed that the presence of a low frequency physiological signal detectable in CM before and/or after tic onset. Both continuous and responsive TS DBS may be possible and we have yet to identify optimal candidates for each approach. The responsive DBS strategy, if possible, offers possible advantages in reducing side effects and using less energy, however it also introduces the disadvantage of a long ramp time, which may negate the possibility of employing therapy prior to tic onset. Improvements in technology which occurred during the course of this study may in the future facilitate a single target ‘sense and stimulate’ approach.

### Supplementary Information

Supplementary Information.

## Data Availability

The datasets generated and/or analyzed during the current study are available in Clinicaltrials.gov. Any other datasets used and/or analyzed during the current study available from the corresponding author on reasonable request.
