# Supplementary material for: Responsive deep brain stimulation for the treatment of Tourette syndrome
Source: Sci Rep. 2024 Mar 18;14:6467. doi: 10.1038/s41598-024-57071-5 (PMC10948908; doi:10.1038/s41598-024-57071-5)
Supplement: Supplementary file 1 — Supplementary Information. [file 41598_2024_57071_MOESM1_ESM.docx]

**Supplementary Table 1.** General adverse events

| **Adverse Event** | **Events** | **Subjects** |
| --- | --- | --- |
| Acid reflux | 1 | 1 |
| Acne | 1 | 1 |
| Akathisia | 2 | 1 |
| Allergy symptoms | 1 | 1 |
| Anxiety symptoms | 3 | 4 |
| Baclofen overdose (accidental) | 1 | 1 |
| Bladder prolapse | 1 | 1 |
| Cellulitis | 1 | 1 |
| Cervical Dystonia (worsened) | 1 | 1 |
| Chest congestion | 1 | 1 |
| Concussion (car accident) | 1 | 1 |
| Crying (uncontrolled) | 1 | 1 |
| Dehydration | 1 | 1 |
| Depressive symptoms | 3 | 3 |
| Electric shock feeling on left temple (transient) | 1 | 1 |
| Esophageal ulcers | 1 | 1 |
| Excess salivation | 1 | 1 |
| Fall | 1 | 1 |
| Fatigue | 1 | 1 |
| Flushing sensation | 1 | 1 |
| Gall bladder inflammation | 1 | 1 |
| Head pain at incision site (chronic) | 1 | 1 |
| Headache | 8 | 6 |
| Headaches (mild post surgical) | 5 | 4 |
| Hiccups | 1 | 1 |
| Hip pain | 1 | 1 |
| Hypomania | 1 | 1 |
| Increase in tics as battery depleted | 1 | 1 |
| Insomnia | 1 | 1 |
| Menstrual bleeding (breakthrough bleeding) | 2 | 1 |
| Motor tic worsening (transient) | 4 | 4 |
| Nausea | 7 | 4 |
| Neck pain /dystonia (worsened) | 1 | 1 |
| Nightmares | 1 | 1 |
| Numbness (Right leg) | 1 | 1 |
| Numbness in right leg | 1 | 1 |
| OCD related tics increased | 1 | 1 |
| Oral thrush | 1 | 1 |
| Ovarian cysts | 1 | 1 |
| Panic attacks | 1 | 1 |
| Psychiatric inpatient hospitalization | 2 | 1 |
| Psychosis symptoms (transient) | 1 | 1 |
| Rash (generalized) | 1 | 1 |
| Rectal Bleeding | 1 | 1 |
| Rectal Prolapse | 1 | 1 |
| Respiratory infection | 2 | 2 |
| Shortness of breath | 1 | 1 |
| Sinus cavity surgery | 1 | 1 |
| Sinus symptoms | 4 | 4 |
| Sleep disturbance | 1 | 1 |
| Slurred speech | 1 | 1 |
| Stomach ulcers | 1 | 1 |
| Stomach virus | 1 | 1 |
| Suicidal Ideation | 2 | 1 |
| Tingling in left hand | 1 | 1 |
| Uterine Prolapse | 1 | 1 |
| Vomiting | 7 | 6 |
| Warm sensation near battery pack (transient) | 1 | 1 |
| Weight gain | 1 | 1 |

**Supplementary Table 2. Adverse Surgical Effects**

| **Surgical Adverse Event** | **Events** | **Subjects** |
| --- | --- | --- |
| Battery depletion and tic worsening | 1 | 1 |
| Edema around right impulse generator (transient) | 1 | 1 |
| Edema at impulse generator | 1 | 1 |
| Edema behind right ear (transient) | 1 | 1 |
| Edema in left neck | 1 | 1 |
| Edema in right foot | 1 | 1 |
| Edema right frontal scalp (transient) | 1 | 1 |
| Edema right impulse generator (transient) | 1 | 1 |
| Erosion left parietal region (repaired) | 1 | 1 |
| Extension cable short circuit (replaced) | 1 | 1 |
| Infection impulse generator | 1 | 1 |
| Infection of depth and strip leads in left brain | 1 | 1 |
| Inflammation at impulse generator site (transient) | 1 | 1 |
| Numbness in left scalp post-surgery (transient) | 1 | 1 |
| Pain (incision site) posterior to the right ear | 1 | 1 |
| Pain (irritation impulse generator) | 1 | 1 |
| Pain (neck) post-surgery | 1 | 1 |
| Pain (tenderness) over impulse generator (transient) | 1 | 1 |
| Pain/sensation (tickling) over left impulse generator | 1 | 1 |
| Scar tissue with tightness in neck | 3 | 1 |
| Shifted impulse generatorin the chest | 1 | 1 |
| Short circuit in right extension cable intermittent | 1 | 1 |
| Soreness in neck | 1 | 1 |
| Stiffness in neck following Botulinum toxin injections | 1 | 1 |

**Supplementary data on subject history, programming, and outcomes.**

**Subject 1:**

Subject 1 was a 23-year-old woman who manifested long duration dystonic tics associated with full arm extension, shoulder jerks, neck twisting, grimacing, forceful upward eye movements, barking, and occasionally groans. A majority of her tics were lateralized to the right side of her body. She rarely manifested simple motor tics and the longer lasting complex motor tics were more consistently present in the recorded thalamic CM region physiology. Simple motor tics were more consistently reflected in cortical recordings. For complex motor tics, the CM region was observed to manifest low-frequency power bands (1-10 Hz) as the discriminating feature for tic. Cortical beta power was identified as a potential physiological marker of tic, however tic movements could not be differentiated from voluntary movements. Closed loop features were tested acutely in the laboratory using the NEXUS-D external interface. Ramp up and down times were tested for tolerability. Subject tolerated a ramping down time of 1sec, but did not tolerate a ramping up time lower than 4 secs. Chronically, the subject remained in an open loop DBS configuration, and had a greater than 30% improvement at 6 months on the study’s primary outcome variable.

**Subject 2**

Subject 2 was a 25-year-old woman who manifested severe motor tics in multiple body regions. These tics were frequently accompanied by severe vocal tics. The tic phenotypes included cursing, kissing sounds, yelling, blinking, snorting, shrugging, eye rolling, finger tapping, head bobbing and hitting her own face. The predominant tics were facial and vocal. Her tics could be reduced by focusing on a task (e.g., singing). The CM region revealed low-frequency power bands (1-10 Hz) associated with her tics. Cortical beta power was identified as a physiological marker of tic, however the marker could not be differentiated from voluntary movement. The closed loop DBS features were tested acutely in the laboratory using the NEXUS-D external interface. Ramp up and down times of 4 sec and 1 sec were selected. Chronically, the subject remained in an open loop DBS configuration and had a greater than 30% improvement at 6 months on the primary outcome variable when programmed and activated on the closed loop setting.

**Subject 3**

Subject 3 was a 33-year-old woman who manifested simple and complex motor tics. Her most common motor tics included changes in facial expression with or without the presence of vocal tics. Included among these tics were sniffing and animal sounds. There were no word vocalizations. She had prominent upper extremity motor tics. The CM region physiology revealed both low (1-10 Hz) and high (30-100 Hz) frequency power associated with motor and vocal tics. This activity was not present during volitional movements. The motor cortex recording revealed a beta power increase (14-24Hz) during tics and voluntary movement. Volitional movements were not appreciated in the CM recordings. Chronic embedded closed loop detection and stimulation were deployed using cortical beta as the control signal to trigger a train of stimulation. Ramping was set to an 8 second onset and 4 second offset. The ramping values were chosen based on tolerability. The limiting side effect for ramping was a metallic taste in her mouth as well as associated hand and facial tingling. Slower ramping mitigated all the side effects. Chronically, the subject had a greater than 30% improvement at 6 months on chronic embedded closed loop settings.

**Subject 4**

Subject 4 was a 39-year-old man with motor tics. His tics were a combination of simple and complex upper extremity motor tics which manifested side-to-side head jerking, dystonic hand movements and eye rolling. His tics also were associated with changes in facial expression. Subject 4 also had a ‘kicking’ tic when walking. His initial DBS lead implantations did not reveal the CM physiology associated with tics, and he was reimplanted with adjustment in his lead locations (previously described^9^. The reimplanted CM region physiology revealed bilaterally low frequency (1-10 Hz) power which was associated with his motor tics. This activity was not present during volitional movements. The motor cortex recording revealed a beta power decrease. He manifested consistent urges to tic which were reflected as a beta power decrease recorded from the motor cortex. The urges were not reflected in CM physiology. Ramping was set to 8 second onset and 1 second offset. There was no fixed stimulation period. He reported stimulation related tingling in his hand and arm region during ramping. Slower ramping times mitigated these side effects. Chronically, the subject had less than a 30% improvement at 6 months on YGTSS (primary outcome) on closed loop DBS settings however he had a greater than 30% improvement on MRTRS at 6 months on closed loop settings.

**Subject 5**

Subject 5 was a 26-year-old woman with vocal tics as her predominant phenotype. She had outbursts of full word phrases, in addition to vulgar coprolalia. She manifested animal sound vocalizations and stuttering. There were prominent facial tics present including eye rolling. The CM region signal revealed (0-10Hz) low frequency power increases which were associated with both her vocal and facial tics. There was a beta power decrease (22-32Hz) in the motor cortex associated with both facial movement and with tic. The cortical beta power decrease was used as the closed loop DBS control signal. The ramping time was set at 8 seconds, followed by 6 seconds on, and then immediately off (<1 second). The limiting side effect for ramping was facial tingling and the ramping was adjusted to avoid this side effect. The subject had a greater than 30% improvement in the primary outcome variable at 6 months on chronic embedded closed loop settings.

**Subject 6**

Subject 6 was a 45-year-old man who manifested severe motor tics including shoulder jerking, arm extension movements and side to side neck movements. Many of his upper extremity tics were characterized as ‘forceful movements.’ There was a strong broadband power increase (1-40Hz), possibly artifact, present in both the CM electrode and in M1 recordings observed during his tics. There was a very mild suppression of tics when placed on optimized open loop DBS settings. A marker for closed loop DBS could not be identified. For the primary outcome variable, we employed a fixed stimulation duration of 10 seconds with a 4 second ramp up time and immediate ramp down. This was tolerated without side effects. Chronic embedded stimulation in both open and closed loop (tested at 6 months) both revealed a <30% improvement on the primary outcome variable.

**Subject 7**

Subject 7 was a 24-year-old man who manifested prominent vocal and facial tics. He had sniffing, upper extremity jerking, abdominal jerking, eye-rolling, and tongue protrusion. He manifested both vulgar coprolalia and copropraxia. He manifested self-injurious hitting behavior (leg and abdomen). CM recordings revealed a low-frequency power increase (1-10 Hz) associated with both his vocal and his motor tics. There was a broadband increase in power (1-20 Hz and 30-35 Hz) in the motor cortex possibly associated with both movement and with tic. His programming was set to a ramp-up time of 8 seconds, there was no fixed duration set for ramping and the ramp-down time was 5 seconds. He felt a strange sensation in his chin or would feel annoyed if the ramping was any faster. Chronic embedded stimulation in open and closed-loop settings (tested at 6 months) both revealed a >30% improvement. The closed-loop setting was associated with mild subjective reports of improvement until the subject reported forgetting to recharge his neurostimulator. The technology implanted for subject 7 was pre-programmed that closed loop settings would ‘disappear’ if the battery was depleted. Any neglect in charging required an in-person visit for device programming and reactivation.

**Subject 8**

Subject 8 was a 24-year-old man who manifested severe vocal and motor tics including squeaking, yelling, arm twitching, neck twisting, jerking extremities, and leg stomping. He also had self-injurious behavior including skin picking, hitting, hand biting, head banging, and poking. CM physiology revealed a low-frequency power increase of (1-10 Hz) which was associated with motor tics. An infection developed 1 month following lead implantation surgery, resulting in the temporary removal of the left CM lead and the left cortical motor strip. Although the left CM lead was reimplanted once the infection cleared, the cortical strip could not be replaced due to scarring of the meninges.There was a strong broadband power increase (1-40Hz), possibly due to artifact, present in CM. M1 recordings from the right cortical strip showed high power before and after tic onset (1-8 Hz). The subject consistently missed follow-up programming appointments which resulted in multiple delays with the data collection protocol. Closed-loop stimulation could not be implemented, because of lack of compliance with missing visits, compliance with recharging, and loss of the left cortical motor strip. The subject received only chronic open-loop stimulation and reported mild tic suppression when he kept his device adequately recharged and in an activated condition. Open loop stimulation revealed a >30% improvement in tics on the primary outcome variable however we could not test closed loop settings.

**Subject 9**

Subject 9 was a 21-year-old man who manifested severe and frequent motor and vocal tics including loud screeches, body twisting, shoulder shrugging, eye rolling, abdominal jerking and leg stomping. He had self-injurious behavior including skin picking, hitting, and head banging. CM physiology revealed a low-frequency power increase of (1-10 Hz) which was associated with motor tics. A low-frequency power increase (1-10 Hz) was also observed in the motor cortex associated with tics, as well as a beta power increase before and after tic onset (20-35 Hz). Empirical open-loop DBS programming did not reveal consistent suppression of tics. He was followed for 24 months and persisted in manifesting less than 30% improvement in tic symptoms at 6 months (primary outcome) and long-term. The optimal open-loop tic setting was a double negative bipolar configuration, which was not sense-friendly, and prevented the initiation of embedded closed-loop stimulation.

**Subject 10**

Subject 10 was a 41-year-old woman who manifested frequent vocal and facial tics. The prominent tics included forced blinking, eye-rolling, eye darting, tongue protrusion, back arching, shoulder movements, and abdominal jerking. She manifested repetitive low squeaking noises, loud screeching, coprolalia, and copropraxia. CM physiology revealed a low-frequency power increase (1-18 Hz) associated with motor tics. Motor cortex recordings indicate a moderate power increase (5-28 Hz) associated with facial tics. Initially the first few post-operative months her tics improved, however, the tic suppression faded and by month-6 there was less than 30% improvement. Closed-loop DBS was not initiated until optimal open-loop stimulation was reached. Ramping was set to a ramp up time of 1sec (2-3.4mA). The baseline amplitude was set to 2mA at all times because ramping without this strategy worsened pain in the left hand. Chronic embedded closed loop DBS revealed a <30% improvement on the primary outcome variable.
